# User preferences on long-acting pre-exposure prophylaxis for HIV prevention in Eastern and Southern Africa: a scoping review

**DOI:** 10.1186/s12889-025-23529-y

**Published:** 2025-07-03

**Authors:** Brian Pfau, Arden Saravis, Sarah N. Cox, Linxuan Wu, Rachel Wittenauer, Emily Callen, Cory Arrouzet, Monisha Sharma

**Affiliations:** 1 Department of Epidemiology, University of Washington, Seattle, WA USA; 2 Department of Global Health, University of Washington, Seattle, WA USA; 3 Department of Pharmacy, University of Washington, Seattle, WA USA

**Keywords:** Pre-exposure prophylaxis (PrEP), HIV prevention, Long-acting formulation, Preference.

## Abstract

**Background:**

Novel formulations for pre-exposure prophylaxis (PrEP) such as injectables, implants, and intravaginal rings are emerging as long-acting alternatives to daily pills for the prevention of HIV. Eastern and Southern Africa (ESA) has the highest HIV burden as well as the highest PrEP coverage globally. To maximize uptake and population health benefits, it is crucial to understand the product preferences of potential users in ESA.

**Objective:**

To conduct a scoping review focused on ESA to understand which PrEP products, particularly long-acting formulations, different subpopulations prefer and factors influencing preferences.

**Design:**

We searched Pubmed, Embase, and conference abstracts using relevant search terms for studies conducted between 2014 and 2024. Studies were eligible for inclusion if they evaluated preferences for at least one long-acting or on-demand PrEP product among potential users in ESA.

**Results:**

We identified 49 studies meeting eligibility criteria. Overall, most participants preferred longer-acting products over oral pills. On-demand PrEP was commonly preferred over daily dosing, and long-acting products were preferred over on-demand dosing. Most studies found injectables to be preferred over daily oral PrEP, implants, and rings, which was observed across subpopulations including men and women, youth, men who have sex with men, and female sex workers. Duration, efficacy, and discretion were the three most important factors influencing participants’ choices.

**Conclusions:**

Long-acting PrEP products, particularly injectables, are preferred by a wide range of individuals in ESA over daily oral pills. Some subgroups preferred monthly oral PrEP or implants citing fear of injections, side effects and stigma-inducing injection marks, emphasizing the benefit of providing multiple products to maximize coverage.

**Strength and limitations of this study:**

Some key populations, such as transgender women, were underrepresented in the literature. Most studies were published before long-acting products’ availability; therefore, they represent hypothetical stated preferences and not real-world uptake.

**Supplementary Information:**

The online version contains supplementary material available at 10.1186/s12889-025-23529-y.

## Introduction

Pre-exposure prophylaxis (PrEP) is a promising HIV prevention product that is highly effective when used with high adherence. Although PrEP uptake has increased significantly since its introduction in 2012, coverage remains below targets, with 1.6 million estimated global users–well below the United Nations goal of 10 million users by 2025 [1]. Additionally, PrEP retention and adherence among those who initiate is suboptimal, reducing its effectiveness [2]. Barriers to oral PrEP uptake and adherence include pill burden, stigma, and lack of discretion associated with oral tablets. In light of these challenges, research has focused on developing several long-acting PrEP formulations, including injectables, implants, and vaginal rings, which could increase PrEP coverage and adherence by providing more convenient and discreet options. Cabotegravir, a bimonthly antiretroviral, was the first long-acting injectable (LAI) approved for clinical use by the US Food and Drug Administration in December 2021 [3]. The intravaginal dapivirine ring (DVR), developed primarily for women in low-income countries, was recommended for use by the World Health Organization (WHO) in January 2021 [4]. An implant with six-month duration is currently in development [5]. Finally, lenacapavir is a first-in-class twice-yearly injectable which has recently demonstrated high efficacy in interim phase 3 clinical trials among both men and women [6, 7]. Further, lenacapavir formulations currently in early trials may extend this duration to a once-yearly injection [8]. In addition to long-acting formulations, oral on-demand PrEP (or event dosing) is an alternative dosing schedule with comparable efficacy to daily use in which patients take a double dose up to two hours before a potential exposure and then once every 24 hours the following two days [9].

Understanding preferences for PrEP products among subgroups with high HIV incidence is crucial for maximizing PrEP coverage and impact. The aim of this scoping review was to synthesize the literature on PrEP perceptions and preferences in Eastern and Southern Africa (ESA), the region most impacted by the HIV epidemic [1]. PrEP is a priority intervention for scale-up in ESA, and policymakers must decide which products to implement and how to tailor demand generation strategies. Understanding the relative preference of LA PrEP compared to oral PrEP by subpopulation is also useful for commodity planning.

## Methods

We conducted a scoping review of peer-reviewed literature on preferences for long-acting PrEP from 2014 to 2024, following Preferred Reporting Items for Systematic Reviews and Meta-Analyses extension for Scoping Reviews (PRISMA-ScR) guidelines [10]. We chose to conduct a scoping instead of systematic review as the latter aims to broadly describe the landscape of a topic, while systematic reviews are narrower, focusing on a specific research question. We conducted a keyword search for relevant articles on PubMed and Embase using the following keywords: “PrEP”, “long-acting”, “discrete choice”, “preferences”, “Africa” (as well as each individual country name), “on demand” (Table S1). We also received grey literature and articles not yet published from a research collaborator, and searched conference literature for relevant findings.

### Inclusion and exclusion criteria

Studies were eligible for inclusion if they met the following criteria: (a) original research; (b) peer-reviewed and published in English between 2014 and 2024; (c) research conducted in ESA; (d) evaluating preferences for at least one long-acting or on-demand PrEP product alongside daily oral PrEP.

### Data screening

Two reviewers (BP and AS) screened the list of references for inclusion into the study. First, titles and abstracts of articles were reviewed and then selected articles underwent full text review. Disagreements were resolved via team discussions. We extracted data on title, publication year, location of data collection, population assessed, main findings, study strengths and limitations. We categorized each study by region and demographic subpopulation of focus.

### Quality assessment

BP conducted a quality assessment of the 49 eligible studies based on generalizability to the target population, participant acceptance rate, and PrEP experience/naïveté of the sample (Table S2).

## Results

### Characteristics of studies

**Fig. 1:**
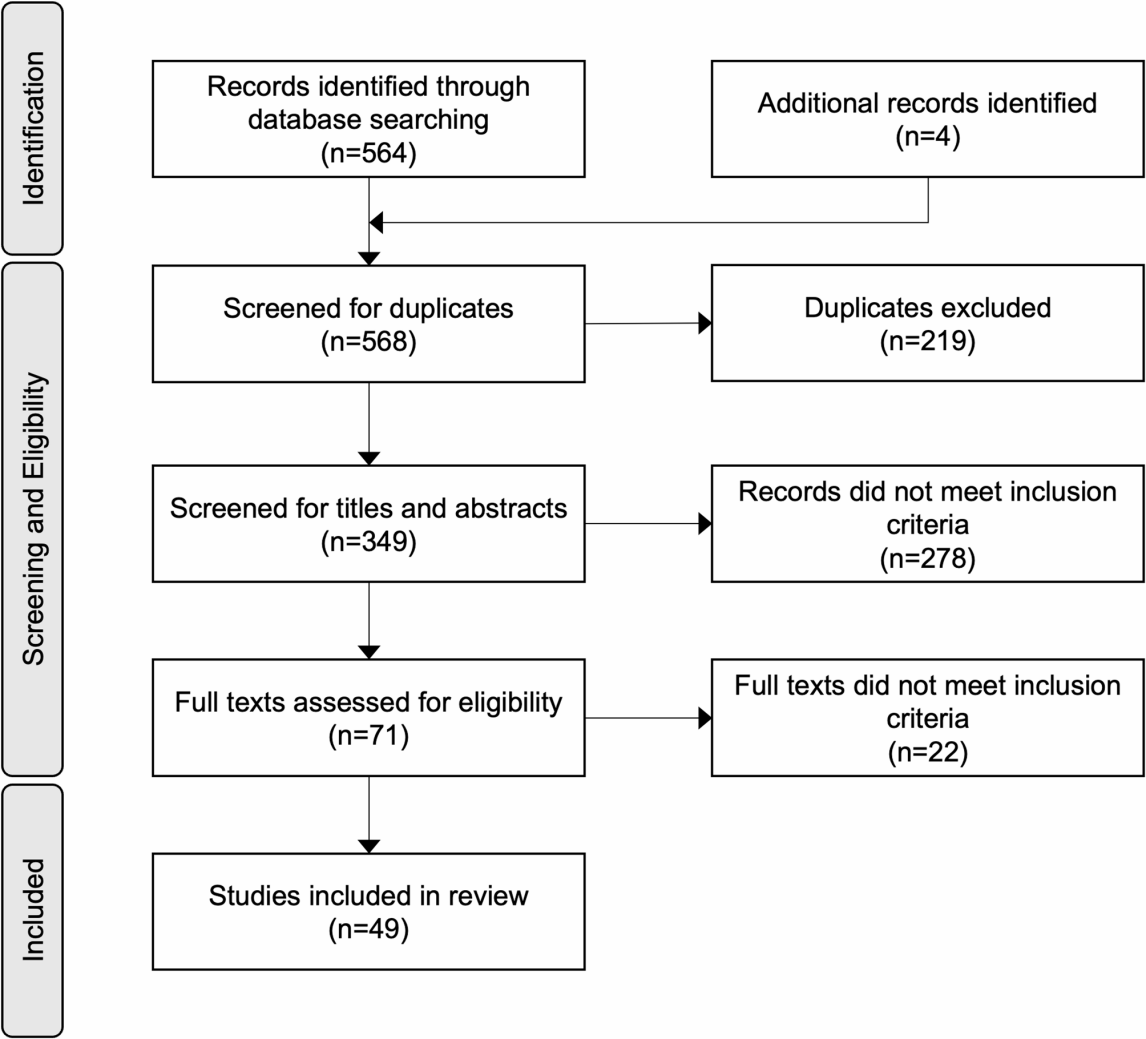
PRISMA flow diagram

Of 349 unique citations identified, 49 articles met eligibility criteria and were included in the review (Fig. 1). Publications were excluded due to lack of relevance, not including comparing daily oral PrEP to a long-acting or on-demand product, or not being conducted in the region of ESA. Study characteristics are summarised in Table 1. Most studies were cross-sectional (29) [11–39] including 13 discrete choice experiments (DCEs) [27–37, 39]. Most or all individuals in 15 studies had prior experience with oral PrEP [11, 13, 15, 20, 21, 23, 33, 40–46], while 12 studies were conducted among exclusively PrEP-naïve participants [12, 14, 16, 17, 24, 28, 31, 39, 47–50] (Table S2). 16 studies were conducted among individuals participating in randomized clinical trials for PrEP [11, 13, 15, 20, 21, 40, 41, 43–46, 51], cohort studies [25, 31, 33], or PrEP implementation projects [49]. Most studies (*N* = 44, 89.8%) included women as they are a priority population for PrEP.

**Table 1:** Characteristics of studies

Characteristics	*N* of studies (%)	
**Formulation**		
Long-acting Injectable (LAI)	36 (73.5%)	
Intravaginal dapivirine ring (DVR)	23 (46.9%)	
Implant	22 (44.9%)	
On-demand	7 (14.3%)	
Other	17 (34.7%)	
**Demographic Subpopulation**		
Men	25 (51.0%)	
ABYM	15 (30.6%)	
MSM¹	3 (6.1%)	
Heterosexual men¹	2 (4.1%)	
Women	44 (89.8%)	
AGYW	27 (55.1%)	
FSW	8 (16.3%)	
Transgender women¹	2 (4.1%)	
Women only	25 (51.0%)	
**Location**		
South Africa	36 (73.5%)	
Kenya	13 (26.5%)	
Uganda	11 (22.4%)	
Zimbabwe	12 (24.5%)	
Tanzania	2 (4.1%)	
Malawi	2 (4.1%)	
Eswatini	2 (4.1%)	
Nigeria	1 (2.0%)	
US	1 (2.0%)	
**UN Sub-region**		
Eastern Africa	26 (53.1%)	
Southern Africa	37 (75.5%)	
**Study Design**		
Randomized controlled trial	10 (20.4%)	
Discrete choice	13 (26.5%)	
Cross-sectional	16 (32.7%)	
In-depth Interview	13 (26.5%)	
Focus group discussion	14 (28.6%)	
**Publication Type**		
Peer-reviewed	39 (79.6%)	
Grey literature	10 (20.4%)	

^1^Study sought to examine this group explicitly.

ABYM: Adolescent boys and young men. MSM: Men who have sex with men. AGYW: Adolescent girls and young women. FSW: Female sex workers

Studies included both men (*n* = 25) [14, 17–20, 22–24, 27–29, 34, 35, 37, 39, 42, 45, 47, 50–56] and women (*n* = 44) [11–17, 19, 21, 23–26, 28, 30–59], including 24 studies which focused on women’s preferences specifically. Seven studies focused on males specifically, including three studies assessing preferences of men who have sex with men (MSM) [18, 42, 55], and two solely among heterosexual adult men [20, 27]. Transgender women were included two studies, one independently and one alongside MSM [42, 58]. Many studies (*n* = 30) included or focused on youth, here referred to as adolescent boys and young men (ABYM) or adolescent girls and young women (AGYW) [14, 15, 17, 19–22, 24–26, 28–32, 37–39, 43–45, 47, 48, 50–53, 55, 57]. Female sex workers (FSWs), a priority population vulnerable to HIV acquisition [1], were assessed in eight studies [16, 26, 30, 37, 47, 48, 55, 57].

Countries in Eastern Africa were represented in 53% of studies while Southern Africa was represented in 76% of studies, including 16 studies (33%) which were conducted in both geographic regions. South Africa was the most represented country (*n* = 36) [11, 13, 17, 19–22, 24, 26–30, 32, 36–38, 40, 41, 43, 44, 46–59], followed by Uganda (*n* = 13) [17, 19–21, 24, 25, 31, 35, 40, 41, 45, 50, 54], Kenya (*n* = 12) [11, 15, 23, 32–34, 36, 42, 45–47, 59], and Zimbabwe (*n* = 11) [13, 17, 19–21, 24, 40, 41, 50, 54, 57]. Nine papers presented findings from three countries combined (Uganda, Zimbabwe, and South Africa) [17, 19–21, 24, 40, 41, 50, 54]. Other papers included Tanzania (*n* = 2) [12, 16], Malawi (*n* = 2) [14, 54], Eswatini (*n* = 2) [32, 39], and Nigeria (*n* = 1) [18].

The majority of studies evaluated LAIs (36) [11–16, 18, 21–23, 25, 27, 28, 31–35, 37–47, 49, 51, 53, 55, 56, 58], followed by the DVR (*n* = 23) [11, 12, 16, 21, 23, 25, 31, 33, 35, 37–41, 43, 44, 46, 51, 53–56], implants (*n* = 22) [18, 22, 23, 26, 28, 30–32, 35, 38–42, 48, 49, 51–53, 57, 59], and other prevention methods such as condoms, films, or gels (*n* = 17) [11, 12, 18, 25, 27, 31, 35, 37, 38, 40–42, 45–47, 53, 58]. All formulations were compared against daily oral PrEP. Seven studies compared preferences between daily oral PrEP and oral on-demand dosing [17, 19, 20, 22, 24, 50], including two studies comparing on-demand dosing to daily oral, injectables, and implants [22, 32]. Table 2 summarises the results of the studies included.

**Table 2:** Summary of studies

Author et al. (Year)^1^	Ref	Subpopulation	Location	Study Design	Formulation	Sample size	Main findings	
Mack et al. (2014)	[47]	Men, Women, AGYW, FSW	South Africa, Kenya	Focus group	LAI, Other	101	1. The majority of FSWs in Kenya preferred LAIs over oral pills or vaginal gels for the convenience of long-term protection and the perception of injections as most discreet. By contrast, few were interested in a gel. Many were interested in the prospect of accepting condomless sex for more money while being protected by PrEP. 2. Preferences among AGYW were equivocal, with some perceiving pills as safer than injections while others perceived LAIs as safer as well as more convenient, long-lasting, and discreet. 3. Members of serodiscordant partnerships strongly preferred LAIs for duration and convenience, though some were hesitant due to fear of needles or injection pain.	
Luecke et al. (2016)	[40]	Women	South Africa, Uganda, Zimbabwe	Interview	LAI, Other, DVR, Implant	68	1. 81% of women preferred long-acting products, citing duration, safety, ease of use, partner concerns, and route of administration. 2. One quarter of women expressed concern about being perceived as HIV-positive if others knew they were taking pills every day.	
van der Straten et al. (2017)a	[41]	Women	South Africa, Uganda, Zimbabwe	Interview, RCT	LAI, Other, DVR, Implant	71	1. When women could select more than one product as “most preferred,” the ring was selected by 94% of women compared with 39% for implants and 33% for LAIs. 2. Participants broadly appreciated continuous protection, discretion, and peace of mind arising from simplified use and infrequent dosing versus worry about forgetting daily doses. 3. Women expressed concern with several formulations about negative reactions from a male partner if he discovered they were using the PrEP product, e.g. feeling the ring, noticing the implant under the skin, or lubrication from the other vaginal formulations.	
van der Straten et al. (2017)b	[46]	Women	South Africa, Kenya	RCT	LAI, Other, DVR	214	1. After one month each of using three different placebo delivery forms for a multipurpose prevention technology (MPT) product, 63% ranked bimonthly injections as most preferred, followed by daily pills (16%) and rings (12%) with 9% preferring condoms. 2. The most cited reason for product choice was ease of use, along with lower dosing frequency among long-acting products and lack of side effects for pills.	
Govender et al. (2018)	[56]	Men, Women	South Africa	Focus group	LAI, DVR	112	1. Gendered focus group discussions revealed conflict between men and women regarding the female partner’s PrEP use. Most women, especially those from rural settings, preferred the LAI for its longer duration, ease of use, and the perceived ability to use it independent of their partner’s support. 2. Most men, however, disapproved of PrEP use in general as they believed that their own partners were faithful and therefore not at risk; taking PrEP would encourage infidelity. Conversely, most women desired a product they could use without their partner knowing, believing that their taking PrEP would license their male partners’ infidelity.	
Krogstad et al. (2018)	[52]	Men, Women, AGYW, ABYM	South Africa	Focus group	Implant	105	1. In a focus group for the design of a long-acting implant, longer duration (≥ 6 months) was a significant attribute among interviewees. 2. Discreetness and comfort were valued with desire for a flexible vs. stiff implant. 3. Biodegradability desired by almost all participants to avoid removal and associated clinic visits.	
Quaife et al. (2018)	[37]	Men, Women, AGYW, FSW	South Africa	Discrete choice	LAI, Other, DVR	661	1. Participants across age and gender broadly valued product efficacy and long-lasting duration, and preferred a multipurpose product for HIV, STI, and pregnancy prevention. 2. Multipurpose prevention was identified as a key consideration for health program design, whether as a hypothetical single product or a bundle of PrEP and contraceptive services, especially for AGYW.	
Siedner et al. (2018)	[39]	Men, Women, AGYW, ABYM	Eswatini	Discrete choice	LAI, DVR, Implant	109	1. 75% preferred a two-month LAI over oral daily. This preference held across sexes, age, education, and sexual behavior.	
van der Straten et al. (2018)	[11]	Women, AGYW	South Africa, Kenya	RCT, Cross-sectional	LAI, Other, DVR	249	1. Formulations most preferred by AGYW were LAIs (62%), followed by oral pills (15%), DVR (12%) and condoms (10%). The most frequently least preferred formulations were the DVR (41%) and oral pills (35%). 2. Between the two countries, South African women had twice the odds of choosing LAIs, while Kenyan women had twice the odds of choosing oral pills.	
Cheng et al. (2019)	[27]	Heterosexual men	South Africa	Discrete choice	LAI, Other	178	1. Among heterosexual men, 48% preferred LAI versus oral (33%) and condoms (20%). 2. Men with children and men who were less risk averse were more likely to prefer LAI. 3. Participants concerned with high likelihood of STIs other than HIV tended to prefer other methods.	
Harling et al. (2019)	[12]	Women	Tanzania	Cross-sectional	LAI, Other, DVR	66	1. While only 5% of respondents had initially heard of PrEP, 79% were somewhat/very interested in LAI, compared to 54% interested in oral daily, 38% in vaginal gel, and 11% in DVR. 2. LAI was ranked most preferred among the four modalities, while 82% picked oral daily as either first or second preferred.	
Kuteesa et al. (2019)	[35]	Men, Women	Uganda	Discrete choice	LAI, Other, DVR, Implant	805	1. Women preferred daily oral, LAIs, and implants similarly but least preferred the DVR. Men preferred oral daily over any other method but least preferred implants.	
Minnis et al. (2019)	[36]	Women	South Africa, Kenya	Discrete choice	LAI, DVR	536	1. AGYW participants strongly valued product efficacy overall and most preferred LAIs. Daily oral pills were least preferred in South Africa, while the DVR was least preferred in Kenya. Many women preferred a potential multipurpose preventive product for HIV and contraception, including a multipurpose vaginal ring.	
Montgomery et al. (2019)	[51]	Men, Women, AGYW, ABYM	South Africa	Focus group, Interview	LAI, DVR, Implant	95	1. Majority of participants interviewed preferred LAI or implants. Many expressed their major influencing factor as efficacy, even if the formulation was expected to be painful. 2. Oral PrEP-experienced men often reported difficulty with the daily dosing regimen, especially on weekends. Some oral PrEP-experienced women felt the pills were more discreet since they do not leave a mark like an injection. 3. Vaginal ring PrEP-experienced women expressed a similar focus on efficacy and expressed concern about discomfort during sex and whether their partner would be able to feel the ring. 4. LAI PrEP-experienced women appreciated the longer dosing frequency and cited side effects as chief complaint, though they perceived the injection to have greater efficacy than other methods.	
Tolley et al. (2019)	[13]	Women	South Africa, Zimbabwe, US	Focus group, RCT, Cross-sectional	LAI	136 (100 African)	1. 93% of non-U.S. participants preferred LAI compared to 64% of U.S. participants. 2. Participants liked the idea that PrEP could be easier to use and of longer duration, though about a third of participants expressed concerns about potential side effects and pain.	
Gill et al. (2020)		Women, AGYW	South Africa	RCT	LAI, DVR	130	1. In the UChoose study, young women were assigned contraceptive methods as a proxy for the DVR, daily pill, or injectables. Participants on the vaginal ring were more likely to request to change to another method than those on injectables (20.7% vs. 1.4%), but those who remained on the ring had higher inherence than those taking daily pills. 2. Participants taking injectables were most likely to report that their method was convenient (96.3%) than those using the ring (83.1%) or daily pills (76.0%). Injectables were most preferred (46.1%), followed by the vaginal ring (37.1%).	
Kidman et al. (2020)	[14]	Men, Women, AGYW, ABYM	Malawi	Cross-sectional	LAI	2089	1. Among 10-16-year-old youth, 80% expressed willingness both to use oral and injectables, but only 52% of girls and 48% of boys would still consider using PrEP if there were side effects. 2. 87% of caregivers indicated that they would want their child to take a daily pill to prevent HIV	
Laher et al. (2020)	[53]	Men, Women, AGYW, ABYM	South Africa	Focus group	LAI, Other, DVR, Implant	68	1. In focus groups, participants expressed preference for long-lasting duration, favoring LAIs and implants for their efficacy, discretion, and duration. 2. Men and women mentioned the difficulty of swallowing large pills as well as concern that others might assume they had HIV if they saw them taking daily pills.	
Minnis et al. (2020)	[28]	Men, Women, AGYW, ABYM	South Africa	Discrete choice	LAI, Implant	807	1. Strong preference for less frequent dosing and for injectables over implants among male and female urban youth including MSM. 2. Participants were willing to trade their preferred formulation for one with a longer dosing frequency.	
van der Straten et al. (2020)	[54]	Men, Women, Heterosexual Men	South Africa, Uganda, Zimbabwe, Malawi	Focus group	DVR	128	1. Pregnant and breastfeeding women were overall accepting of PrEP and highly valued safety for mother and child regarding PrEP use. Some reported fears about miscarriage or birth defects resulting from PrEP use. They stressed the importance of personal choice in HIV prevention and wished to have a variety of options so they could choose the product that worked best for them. 2. Participants feared exacerbation of pregnancy symptoms such as vaginal discomfort with the DVR or nausea for oral pills. Taboos against vaginal insertion or taking medicine during pregnancy could be a barrier to initiation for both formulations. 3. Partner support was paramount, and women typically considered PrEP use to be a decision made in conjunction with their partners.	
Dietrich et al. (2021)	[24]	Men, Women, AGYW, ABYM	South Africa, Uganda, Zimbabwe	Cross-sectional	On-demand	1339	1. Though awareness of PrEP was low, 95.8% of participants were willing to use PrEP. Men were more likely to prefer on-demand (65.2% vs. 53.8%) while women were more likely to prefer daily (46.3% vs. 34.8%). 2. Preference for on-demand was driven by dislike of daily tablets and believing one was not frequently enough at risk to justify daily dosing. Those who preferred daily valued being constantly protected and/or perceived daily dosing to be more effective.	
Little et al. (2021)	[26]	Women, AGYW, FSW	South Africa	Interview, Cross-sectional	Implant	24; 600	1. 78% of AGYW and FSW said that they would be likely or very likely to use a hypothetical implant. 2. Participants preferred an implant with minor insertion pain and mild side effects, a 24-month product over 6 month, and one that was biodegradable and did not need to be removed.	
Malone et al. (2021)	[38]	Women, AGYW	South Africa	Discrete choice	LAI, Other, DVR, Implant	1002	1. AGYW preferred a 3-month injection overall, followed by a monthly pill, over a 2-month or 6-month injection. LAIs were preferred over implants and vaginal rings. 2. Participants most preferred to receive PrEP from a nurse in a mobile clinic over other settings.	
Montgomery et al. (2021)	[29]	Men, ABYM	South Africa	Discrete choice	LAI	406	1. Young men reported several features to be “very important”: perceived efficacy (94%), where one has to go to get it (88%), dosing frequency (87%), and removability in the event of side effects (85%). 2. Both MSW and MSM valued privacy and discretion, though more MSW valued being able to use PrEP without their partner knowing (46%) compared to MSM (27%). 3. 94% reported willingness to pay for an LAI.	
Ngure et al. (2021)	[15]	Women, AGYW	Kenya	RCT, Cross-sectional	LAI	350	1. Among young women exiting the MPYA PrEP monitoring study, 36% preferred injectable CAB-LA, 34% preferred daily pills, 22% preferred implants, 15% preferred vaginal rings. 2. No association between preference and age, HIV risk, marital status, contraceptive method among those using contraception, or other variables.	
Nkomo et al. (2021)	[57]	Women, AGYW, FSW	South Africa, Zimbabwe	Focus group	Implant	110	1. Adult women including FSWs were interviewed about an MPT implant to act as HIV prevention and as a contraceptive. They desired a biodegradable implant with as long a duration as possible, yet that could be easily removed if needed, such as for desire to return to fertility. Scarring and side effects were concerns for both implant-experienced and inexperienced women. An MPT with independently removable PrEP and contraceptive components was ideal for many FSWs who were concerned about irregular bleeding from hormonal contraceptives.	
Poteat et al. (2021)	[58]	Transgender women	South Africa	Interview	LAI, Other	36	1. Transgender women in interviews were highly aware of PrEP (32/36 participants) and one quarter had taken it or were currently taking it. Most preferred LAIs while some preferred a topical product, but the importance of product choice was salient. Dismay over daily dosing was the most common reason for preferring LAIs or a topical product over oral pills. 2. Due to pervasive discrimination in healthcare settings, many trans women were enthusiastic at the prospect of only needing to visit a healthcare provider every six months vs. monthly for oral pills.	
Beckham et al. (2022)	[16]	Women, FSW	Tanzania	Focus group, Interview, Cross-sectional	LAI, DVR	496; 10; 20	1. Where 92% of FSWs participating were initially unaware of PrEP, 88% preferred LAI over oral daily, citing dosing frequency, discretion, and belief in higher efficacy. 2. 58% felt PrEP was personally worth it to take, and those who did were more likely to have recent STI symptoms or diagnosis. 3. Many FSWs stressed that they would not reduce condom use if they took PrEP.	
Brown et al. (2022)	[48]	Women, AGYW, FSW	South Africa	Interview	Implant	36	1. In qualitative interviews, end-users generally perceived some drawbacks of the implant such as side effects and pain during insertion but believed these were outweighed by the benefits of a highly effective and long-lasting PrEP product. 2. HCPs acknowledged concerns about increased condomless sex but recognized value in the implant’s extended protection and improved adherence compared to oral PrEP. 3. Despite low awareness of oral PrEP, end-users expressed willingness to try a PrEP implant. FSWs liked the ability to protect themselves from HIV without requiring a client to use a condom.	
Dietrich et al. (2022)	[17]	Men, Women, AGYW, ABYM	South Africa, Uganda, Zimbabwe	Cross-sectional	On-demand	1330	1. 60% stated a preference for on-demand, with males and older young adults more likely to prefer on-demand. 2. Preference for on-demand PrEP decreased with more frequent sexual activity in the last month.	
Gates Foundation (2022, not published)	[59]	Women	South Africa, Kenya	Focus group	Implant	32	1. In an assessment of potential combination PrEP and contraceptive methods, implants were perceived as invasive and too visible to be discreet, and other options were perceived as more convenient. 2. Separate coadministered LAI and contraceptive injections were acceptable as long as both injections were of the same duration.	
Little et al. (2022)	[30]	Women, AGYW, FSW	South Africa	Discrete choice	Implant	600	1. 78% of respondents stated they would be likely or very likely to use an implant if one were available. 82% expressed preference for a dual use product for PrEP and contraception. 2. Broad preference for 24-month vs. 6 month protection interval and for a biodegradable (dissolvable) product.	
Mayanja et al. (2022)	[31]	Women, AGYW	Uganda	Discrete choice	LAI, Other, DVR, Implant	285	1. 47.6% preferred oral PrEP, 52.4% preferred hypothetical PrEP alternatives. 2. Preference for oral PrEP was associated with 50% higher PrEP uptake. 3. Low awareness of oral PrEP (24.5%), and even lower awareness of hypothetical alternatives (LAI 4.2%, DVR 2.3%, HIV vaccine 1.5%, implant 0%).	
Ogunbajo et al. (2022)	[18]	MSM	Nigeria	Cross-sectional	LAI, Other, Implant	305	1. 88% of MSM, were willing to use LAI with 44% of participants preferring it. 21% preferred daily oral, 17% preferred lubricants, 10% preferred all formulations equally, while only 6% preferred implants. 2. Those who preferred LAI were more likely to be single, report inconsistent condom use, and report having a primary care provider.	
Webb et al. (2022)	[19]	Men, Women, AGYW, ABYM	South Africa, Uganda, Zimbabwe	Cross-sectional	On-demand	1330	1. PTSD symptoms were not associated with willingness to take PrEP or preference for on-demand vs. daily PrEP.	
Bailey et al. (2023)	[42]	MSM, Transgender women	Kenya	Interview	LAI, Other, Implant	423	1. In pairwise comparisons, quarterly injections were most preferred (26%), followed by monthly pills (23%), a yearly implant (19%), condoms (12%), and oral daily (1%). 2. When “forced” to choose the most preferred product, 37.1% preferred a quarterly injection, 34.8% preferred a monthly pill, 25.8% preferred a yearly implant, and 2.4% preferred oral daily. 3. Transgender women were more likely to prefer the implant over quarterly injections than gay or bisexual men. 4. Muslim participants had considerably greater preference for implants compared to Christians, adherents of African indigenous religions, and religious unaffiliated participants.	
Dietrich et al. (2023)	[50]	Men, Women, AGYW, ABYM	South Africa, Uganda, Zimbabwe	Focus group, Interview	On-demand	189	1. In the CHAPS study, conducted before the widespread regional rollout of PrEP, both male and female participants who preferred daily PrEP over on-demand desired continuous protection in the event of unplanned sexual activity, whether consensual sex or in case of sexual violence. Many also perceived the daily regimen to be more effective overall. 2. Participants who preferred on-demand dosing feared stigma associated with taking daily ARVs, as well as adherence concerns such as pill fatigue. Many felt they were not frequently sexually active enough to warrant a daily pill, and expected fewer side effects compared to daily.	
Fynn et al. (2023)	[44]	Women, AGYW	South Africa	Focus group, RCT	LAI, DVR	33	1. In focus group discussions for the UChoose study (See above), though most participants were initially hesitant to try a new method, they were nevertheless encouraged to have a diversity of options to find the product that worked best for them. 2. Injections were most popular, followed closely by the DVR, chiefly due to less frequent dosing and ease of use.	
Jansen van Vuuren et al. (2023)	[49]	Women	South Africa	Interview	LAI, Implant	425	1. Women were most likely to prefer the same PrEP formulation as that which they were using or had used for contraception.	
Kakande et al. (2023)	[20]	Men, ABYM, Heterosexual Men	South Africa, Uganda, Zimbabwe	Interview, RCT, Cross-sectional	On-demand	647	1. 65.2% of participants stated a preference for on-demand PrEP. Preference was higher in Uganda (76.8%) and Zimbabwe (70.4%) than in South Africa (45.5%). 2. Preference for on-demand PrEP increased with age. 3. Reasons for preferring on-demand PrEP included not liking daily tablets (38%), not thinking one is exposed to HIV frequently enough to warrant a daily pill (17%), fear that taking daily PrEP might make others think one has HIV (15%), and concern for pill fatigue (11%). 4. Reasons for preferring daily PrEP included continuous protection (45%), better protection overall (19%), and ease of daily routine vs. remembering to take pill before having sex (15%)	
Little et al. (2023)	[32]	Women, AGYW	South Africa, Kenya, Eswatini	Discrete choice	LAI, Implant, On-demand	1263	1. Women most preferred 12-18-month removable implants followed closely by 3-6-month LAIs and oral on-demand, with daily oral pills, weekly skin patches, and the monthly DVR ranked lowest preferred. There was a strong preference for multi-purpose products which could prevent pregnancy and/or STIs. 2. Efficacy, duration, and reversibility were the most important attributes for implants	
Mataboge et al. (2023)	[55]	Men, Women, AGYW, FSW, ABYM, MSM	South Africa	Focus group	LAI, DVR	109	1. Participants overall preferred LAI (Cabotegravir) over DVR. Most cited reduced time spent at overcrowded clinics, ease of method continuation, and removed burden of daily dosing as reasons for embracing long-acting formulations. 2. Most AGYW and all pregnant AGYW and FSWs would not use the DVR because of perceived side effects, and some ABYM worried about their partner’s potential pain during intercourse and lower efficacy. Participants cited familiarity with injectable contraceptives as part of the acceptability of LAIs. However, some AGYW feared similar side-effects to injectable contraceptives, e.g. menstruation cessation and weight gain.	
Ngure et al. (2023)	[21]	Women, AGYW	South Africa, Uganda, Zimbabwe	RCT, Cross-sectional	DVR	247	1. Similar proportions of preference between the ring (38.1%) and oral PrEP (40.5%), with 19% preferring both equally.	
Tran et al. (2023)	[34]	Men, Women	Kenya	Discrete choice	LAI	50	1. HIV + participants generally preferred hypothetical long-acting formulations to their current ART therapies.	
Wara et al. (2023)	[33]	Women	South Africa, Kenya	Discrete choice	LAI, DVR	394	1. 75% of participants preferred a potential LAI over daily oral PrEP. 2. In South Africa, longer duration of effectiveness was the major factor influencing preference (87% South Africa vs. 42% Kenya). Discretion was a larger factor in Kenya (5% South Africa vs. 49% Kenya). 3. Conversely, 87% of participants preferred oral PrEP over a vaginal ring mostly due to concern about discomfort (82% South Africa, 48% Kenya). 4. Preferred frequency of PrEP use by ranking order was once a year (31%), once a month (16%), once every 2–3 months (15%), before sex (13%), every day (12%) and once every six months (11%)	
Were et al. (2023, not published)	[23]	Men, Women	Kenya	Focus group, Interview, Cross-sectional	LAI, DVR, Implant	6013; 257	1. 91.4% of participants willing to use LAI preferred a six-month duration over a two-month. 63.6% preferred subcutaneous injection over intramuscular injection and 92.4% preferred provider-administration over self-administration. 2. 41.5% of participants willing to use implants preferred a biodegradable that did not need to be removed, while 58.5% preferred a nonbiodegradable because it could be removed if needed and/or because they feared its absorption into the body. 3. Among those who declined PrEP, most cited reasons were the perceived burden of taking a daily pill (35.4%), fear of side effects (21.2%), already consistently using condoms (9.9%) and trusting their partner (9.5%). 4. Participants desired to use PrEP if alternative formulations were available without side effects or the burden of a daily pill and its associated stigma.	
Kamya et al. (2024)	[45]	Men, Women, AGYW, ABYM	Kenya, Uganda	RCT	LAI, Other	984	1. Participants in the SEARCH study received a dynamic choice prevention (DCP) intervention, enabled to choose between oral PrEP, PEP, and CAB-LA and freely switch between or stop products based on product preference or period of risk. Compared to standard-of-care, biomedical covered time for DCP participants was 65.6% and 52.8% higher for men, and women, respectively. 2. 56% of participants receiving the DCP intervention used CAB-LA, while 53% used daily oral PrEP. 42% of CAB-LA users were not using any PrEP product in the month prior to the study, suggesting that CAB-LA could expand uptake to potential users who would otherwise not be amenable to using PrEP.	
Mayanja et al. (2024)	[25]	Women, AGYW	Uganda	Interview, Cross-sectional	LAI, Other, DVR, Implant	265	1. AGYW most commonly preferred an HIV vaccine (34.7%) followed by daily oral pills (25.7%), LAIs (24.9%), implants (13.6%) and the DVR (1.1%). Preference for LAIs and for implants increased with age but was lower for women with partners with positive or unknown HIV status. 2. In qualitative interviews, ease of use and familiarity with pill taking motivated many who preferred oral pills, but others were dissuaded by pill burden, lack of discretion, perceived stigma, and side effect concerns.	
Mthimkhulu et al. (2024)	[22]	Men, ABYM	South Africa	Cross-sectional	LAI, Implant, On-demand	145	1. Most men would consider using were a monthly pill (74.6%), the implant (62.7%), oral on-demand (59.2%), and the six-month LAI (57.7%). 50% would consider oral daily, and 43.7% would consider a bimonthly LAI. However, in group interviews men generally agreed that any product would be acceptable as long as it was effective. 2. If only one choice were available, the most preferred options were a monthly pill (31.7%), the six-month LAI (28.2%), and the implant (19.7%). 3. Side effects were the primary concern around PrEP, especially sexual/fertility issues and discomfort on implant insertion.	

^1^Articles are listed by year, then alphabetically

### Preferred products by subpopulation

#### Women

Adult women and adolescent girls and young women (AGYW) most commonly preferred LAIs over other long-acting products as they were perceived as having greater efficacy and longer duration, were suitably discreet, and required little or no partner involvement [56]. Women participating in the CAPRISA 082 study in South Africa who had previously used an implantable or injectable contraceptive were more likely to choose that method for PrEP [49]. In a South African focus group, despite overall preference for LAIs over oral pills and the DVR, a few AGYW were dissuaded from injectable PrEP for fear of side effects similar to injectable contraceptives, such as menstruation cessation and weight gain [55]. However, LAIs were not universally preferred. In a case crossover study providing three contraceptive methods (daily oral, injectable and NuvaRing) to adolescent girls aged 15–19 in South Africa, participants were asked their preference for potential HIV prevention method: 46% chose injectable, 33% ring, and 10% daily pill [43]. Similarly, in a DCE of fishing communities in Uganda, women reported an equal preference for implants, injectables, and oral PrEP, although the ring had a negative preference [35]. In another study of AGYW in Kampala, Uganda, preferred methods were: HIV vaccine (34%), oral PrEP (26%), injectable (25%), implant (14%), and vaginal ring (1%); interest in injectable PrEP was higher for older AGYW and those with STIs [25].

Interest in the DVR was generally low outside of studies among women participating in DVR clinical trials. In the ASPIRE study, a multinational clinical trial of the DVR, ,94% of women selected the ring as their most preferred product, compared with 39% for implants and 33% for LAIs [41]. However, many women expressed concern about discretion, such as whether their partner could feel the ring during intercourse or whether it could come out accidentally [51]. However, in most other studies, DVR was less frequently preferred compared to LAIs [12, 33, 51, 55]. Most AGYW in one focus group would not use the DVR due to perceived side effects, especially pain during intercourse, as well as concern for hygiene and use during menstruation [55].

Women reported interest in using implantable PrEP and stated that benefits of an effective and long-lasting product outweighed potential drawbacks such as side effects or pain on insertion. They also expressed interest in a dual use product for PrEP and contraception in surveys and interviews. Since implants are already a common modality for extended-release contraceptives, a device which combines the two could be attractive to women seeking protection from both HIV and pregnancy [18, 23, 28, 30, 32, 42, 48, 51].

In a study among pregnant and postpartum women participating in the PrEP-PP and PrIMA-X trials in South Africa and Kenya, respectively, many voiced safety concerns for mothers and infant during pregnancy and breastfeeding. These women, most of whom had recent experience with oral PrEP, tended to prefer long-acting products over daily oral for their longer duration (especially in South Africa) and increased discretion (especially in Kenya) [33]. All pregnant AGYW interviewed in a study in South Africa would not use the DVR due to perceived side effects [55]. Pregnant and postpartum women in multi-national focus groups mentioned fears of side effects ranging from exacerbation of pregnancy discomfort to severe outcomes such as miscarriage or birth defects. However, they were overall accepting of PrEP as long as it was safe and effective, and stressed the importance of choice and the ability to choose the formulation that worked best for them [54].

#### Female sex workers (FSWs)

Awareness of PrEP was low among FSWs in South Africa [48] and Tanzania [16], but after learning about PrEP most participants were willing to use it, particularly those who had recent symptoms or diagnosis of a sexually transmitted infection (STI). Many FSWs stated that protection by PrEP could enable them to have sex with clients without requiring condoms [48] or to accept condomless sex at a higher price [47], highlighting potential concerns regarding risk compensation. However, others viewed PrEP as a protective complement to condoms and stated that they would continue to use condoms to prevent STIs and pregnancy [16]. 86% of FSWs responded that they would likely use an implant in a South African DCE [30]. This high proportion of acceptability was complemented by qualitative interviews in South Africa [48], where FSWs commended the benefit of continuous protection and noted that it would be worth a brief amount of pain during insertion. Similarly, 88% of Tanzanian sex workers preferred LAIs over oral daily, although a smaller majority (58%) felt PrEP was worth taking. LAIs were considerably preferred over the DVR in interviews with South African FSWs; all sex workers stated they would not use the DVR due to perceived side effects and concern that a client might notice the device [55]. A DCE among FSWs in South Africa found strong preference for LAI with negative preference for DVR and microbicide gel [37].

#### Men

MSM tended to prefer LAIs over implants or oral pills. Among Nigerian MSM, preference for LAIs was associated with single relationship status, inconsistent condom use, and having a primary care provider [18]. Duration/dosing frequency was a highly prioritized product attribute for male participants. Oral PrEP-experienced young men in South Africa reported difficulty with daily dosing [51]. Men of all orientations valued privacy and discretion, though more men who have sex with women (MSW) valued being able to use PrEP without their partner knowing compared to MSM [29].

Preferences among heterosexual men were specifically assessed in two studies [20, 27]. In a survey of urban heterosexual men in South Africa, 48% preferred LAIs compared to 33% who preferred oral and 20% who preferred condoms alone. Men who had children or who were less risk-averse were more likely to prefer LAIs. Men concerned with high likelihood of STIs other than HIV were more likely to prefer condoms over LAIs alone. As choices were discretely ranked, it was not clear how many men who preferred condoms for their protection against other STIs would prefer to use LAIs and condoms in combination [27]. In a mixed methods study conducted in South Africa, Uganda, and Zimbabwe among participants in the CHAPS trial, a majority of heterosexual male youth (65%) preferred on-demand oral PrEP compared to daily dosing. Those who did not believe they were exposed to HIV regularly enough to warrant taking a daily pill tended to prefer on-demand dosing in qualitative interviews [20]. 

#### PrEP-naïve participants

Participants who had not previously used PrEP and were participating in a PrEP clinical trial tended to prefer long-acting products over daily pills for their longer duration and less frequent dosing [12, 14, 16, 17, 24, 28, 31, 39, 47–50]. While daily dosing was highly acceptable to most participants in studies allowing multiple selections, long-acting products and especially injectables were consistently preferred [12, 14, 24].

### Product attributes driving preference

#### Duration

Product duration (dosing frequency), was the most important factor driving user preferences in many studies across subpopulations [13, 28, 29, 33, 51]. Oral PrEP-experienced pregnant and postpartum women most commonly cited product duration as a factor for switching to LAI [33]. Oral PrEP-experienced men also frequently reported difficulty adhering to a daily dosing schedule, especially on weekends, as well as difficulty swallowing the pill [51] (the most common oral PrEP formulation, emtricitabine-tenofovir, is a large tablet [19 mm] which can be difficult to swallow even for users who take other smaller tablet medications.) Male and female youth in a South African DCE valued product duration highly and were typically willing to trade their preferred product for one with a longer dosing frequency [28].

Notably, some youth in interviews in South Africa suggested an ideal formulation as a monthly rather than a daily pill [55]. This would reduce the burden of daily dosing for users who preferred an oral tablet over other long-acting formulations, such as pain associated with injections.

#### Efficacy

Many participants emphasized PrEP efficacy as a driving factor of product preference [36]. They noted the difficulty of adhering to a consistent dosing schedule, which hinders the observed effectiveness of oral PrEP. PrEP effectiveness was the strongest factor driving preference in several studies, commonly among pregnant and postpartum women [33] and among youth [29, 51]. High PrEP effectiveness outweighed participant concerns about side effects such as pain upon injection or product insertion.

#### Discretion

Discretion (i.e., being able to use a PrEP formulation without a partner or the community knowing) was a commonly mentioned concern, particularly among women and persons who did not wish to disclose their PrEP use to their partners. In several studies evaluating oral PrEP alongside LA formulations, including women [41], FSWs [16], and adults [23], participants expressed concern about stigma associated with daily pill use. They feared others would think they had HIV upon discovering that they were taking a daily antiretroviral pill. Similarly, a visible preventive product could be seen as a mark of sexual indiscretion or promiscuity, as voiced in one South African focus group including participants of all genders and sexual orientations [53]; participants stated using PrEP could sow distrust or signal infidelity to romantic partners, and a woman who used PrEP might be seen as sexually promiscuous.

Opinions differed regarding discretion of different products. While many participants found daily pills indiscreet, others felt it was the most discreet since it does not leave a mark like an injection [51]. Others, especially women, were concerned about their partner knowing about their PrEP use. This is echoed by qualitative interviews among men in South Africa who stated they would not be supportive of any type of PrEP use in their female partners as it may be indicative of infidelity [56]. Many participants expressed disinterest in the DVR for fear that their partner would feel the ring during intercourse, or that it might fall out and cause embarrassment. Concerns about partners noticing signs of PrEP use were also mentioned relating to the implant; many interview participants disliked the notion of a visible device under the skin and preferred one that would not be seen by others.

#### Implant biodegradability

Overall, participants preferred biodegradable implants which would not need to be removed by a provider, alleviating the need for an extra clinic visit and pain during removal [30, 52] [60]. However, in one study in Kenya, many participants (especially FSWs) preferred a nonbiodegradable implant for its reversibility, as it could be removed if needed. Some also expressed fear about effects of degraded materials being absorbed into the body [23].

#### Logistical challenges

Long-acting formulations overcame challenges of frequent visits to clinics or pharmacies, which participants described as overcrowded, lacking in privacy, and inconvenient or inaccessible [51, 55]. These challenges drove preference for longer-duration products and for biodegradable implants, requiring fewer visits.

#### Injection fear

A common barrier to LAI acceptability was dislike/fear of needles. The current formulation of cabotegravir is injected in the buttock, but both males and females in South Africa tended to dislike this location for fear of discomfort especially while sitting [28, 47]. Some young women were uncomfortable having to disrobe to receive an injection in the buttocks [47].

#### Side effects

Fear of real or perceived side effects was a salient factor influencing preference, especially for younger participants–about half of male and female youth in a study in Malawi reported being unwilling to use PrEP if they experienced side effects. A DCE in South Africa found that the absence of side effects was important to males but not females [37]. Pain at injection site was the most frequently mentioned concern of LAIs, although youth in qualitative interviews stated that efficacy was a more significant factor even if the formulation was expected to be painful [29, 51]. Similarly, pain upon insertion was a concern regarding implants, but overall participants felt that benefits of an effective and long-lasting PrEP product outweighed potential for pain. Qualitative interviews indicated a preference for a flexible versus a stiff implant for increased comfort.

Pain or discomfort also influenced acceptability of the DVR, especially discomfort during intercourse. Among pregnant and postpartum women in South Africa and Kenya, most of the participants who preferred oral PrEP over a vaginal ring did so due to concern about physical discomfort [33]. In another study among FSWs in Tanzania, participants expressed concerns about infertility associated with the DVR [16]. Conversely, in two studies conducted in Kenya and in South Africa, Uganda, and Zimbabwe, participants appreciated the reversibility of DVR and implants, which could be removed if side effects occurred [23, 41].

#### Oral on-demand

Five studies conducted in South Africa, Uganda, and Zimbabwe [17, 19, 20, 24, 50] assessed preference for oral on-demand PrEP compared to daily use, and two studies, one in Eswatini, Kenya, and South Africa [32]and one in South Africa alone [22] compared on-demand PrEP to a long-acting product. Overall, 60% of male youth [17] and 65% of youth MSM [20] preferred on-demand PrEP over daily oral tablets, with older youth tending to have greater preference for on-demand dosing. Having more frequent sexual intercourse was associated with a lower preference for on-demand PrEP and greater preference for daily oral PrEP. Participants who preferred on-demand PrEP cited not liking daily dosing, intermittent/infrequent sexual exposure, stigma, and pill fatigue, and fewer side effects with less frequent dosing [50]. Conversely, those who preferred daily use cited desire for continuous and/or improved protection and comparative ease of use by dosing every day instead of having to remember to take it before sex. One study examined the effect of post-traumatic stress disorder (PTSD) on preference in SSA youth and found no significant association between PTSD symptoms and a preference for on-demand versus daily PrEP, with a 61% vs. 51% preference for on-demand dosing in those with and without PTSD symptoms, respectively [19]. South African men found oral on-demand, LAIs, and implants similarly acceptable, but if only one choice was available, they preferred a once-monthly pill (32%), six-month LAIs (28%) or implants (20%) over on-demand (2%) or a two-month injectable (5%) [22].

## Discussion

This scoping review evaluated preferences and acceptability for various PrEP products among different subgroups in ESA. Overall, we found high acceptability of LA PrEP across participant subpopulations and geographic regions, suggesting that LA modalities can expand PrEP coverage among persons who could benefit. The primary factors driving participant preferences for LA PrEP were efficacy, duration and discretion. Overall, long-acting injectables were most preferred over the other LA products, including implants and the DVR. However, we identified heterogeneity in preferences among subgroups, which suggests that a variety of products will likely be needed to optimize coverage of HIV prevention. While long-acting injectables were preferred by most participants, obtaining maximum PrEP coverage may require offering multiple options. The importance of offering multiple prevention options has been observed in the family planning literature which demonstrates increased contraceptive use with the availability of more contraceptive methods [61].

A previously published review assessed values and preferences for long-acting injectable PrEP; however, most studies included were published on or before October 2021, before regulatory approval of the first long-acting ARV for use as PrEP, CAB-LA [62]. Authors found broad interest and preference for LAIs and highlighted perceived benefits of discretion and less frequent dosing. Our review adds to the literature by evaluating other long-acting modalities in addition to LAIs and including additional publications after the introduction of injectable PrEP. Further, we focus on ESA, as the region with the largest HIV burden globally and the most widespread rollout of PrEP. In ESA, the majority of transmission occurs through heterosexual mixing. Additionally, higher rates of oral PrEP use can influence attitudes among the general population regarding HIV prevention. One example is parental attitudes toward provisioning PrEP for their children. An overwhelming majority of caregivers of adolescents in Malawi (87%) and in South Africa (85%) expressed desire for their children to take PrEP [14, 63]. This can be contrasted with a study in the American south in which parents of LGBTQ adolescents, though generally positive about PrEP, expressed relatively low intention for their children to take it [64].

Interestingly, the dapivirine ring, which was designed for use in low-resource health systems as in ESA, was a less popular choice than other long-acting options even among women with experience using DVR [51]; this was largely due to perceived side effects, especially pain during intercourse, and concern about indiscretion or an impact on the male partner’s pleasure during intercourse. One study within a DVR clinical trial found overwhelming acceptability of the ring after 28 weeks of follow-up, suggesting that the DVR could become more acceptable after experience with use [41]. However, the preferences of women choosing to participate in DVR clinical trials may not be representative of the general population. That said, one study [55] highlighted focus group discussions among women [65] and their male partners [66] that suggest that while a considerable proportion of partners notice the ring during sex, the impact on sexual pleasure for both partners is minimal and in some cases positive.

Across studies, the most commonly reported concern about long-acting PrEP was potential side effects. However, empiric data shows that actual side effects were less frequent or severe than participants anticipated. Side effects of antivirals for PrEP are typically mild and of short-term duration, yet about half of youth participants in a study in Malawi stated they would not consider using PrEP if there were side effects [14]. Similarly, some women who had experienced side effects from injectable hormonal contraception worried about similar effects from injectable PrEP [55], although these have not been observed [67]. Health communication that assuages these fears and increase LAI uptake.

Pregnant and breastfeeding women were particularly concerned about side effects that could harm the fetus or infant, with some expressing fear of miscarriage or birth defects [54]. Though oral PrEP is widely understood to be safe during pregnancy [68, 69], other long-acting formulations have been slower to establish similar safety profiles [70]. As these data emerge, educating pregnant and breastfeeding populations on the safety of these formulations can enable informed decisions regarding PrEP uptake.

Two studies compared oral on-demand PrEP with a long-acting formulation, which both found similar acceptability between on-demand and long-acting products but a notable preference for long-acting products over on-demand dosing [22, 32]. Since participants frequently preferred on-demand over daily dosing, and because the schedule may have similar patient advantages to long-acting formulations (comparable efficacy, longer dosing frequency, increased discretion, etc.), more research is needed to understand preferences.

One study assessed religious background and found a much greater preference for implants among Muslims compared to Christians, adherents of African indigenous religions, and non-religious participants [42]. Factors driving these preferences were not examined. Similarly, ethnicity was generally captured only on the national level in the studies included. As ESA is ethnically and religiously diverse, these and other markers of cultural identity may be associated with different values surrounding sexuality and HIV prevention and could aid in developing culturally appropriate messaging.

Our review highlighted several gaps in existing studies. Only two studies examined preferences of transgender women in ESA, despite their having up to 13 times higher HIV incidence than the general population globally [42, 71]. Transgender women are often grouped with MSM in preference studies, yet their preferences were distinct in the one study comparing the two groups, in which transgender women tended to prefer implants while MSM preferred LAIs. This highlights the importance of assessing heterogeneity in preferences across demographics as preferences of cisgender women or other sexual minority individuals assigned male at birth do not necessarily align with those of transgender women.

We also found generalizability concerns in many publications that assessed PrEP-experienced participants and particularly those participating in PrEP clinical trials. 12 studies were conducted among clinical trial participants; several preference studies were conducted during trial follow-up visits, therefore only participants who continued PrEP were included and preferences of those lost to follow up were not assessed. Findings from these studies may not generalize to those who are PrEP naive or who are lost to follow-up due to adherence challenges. Individuals who experience challenges using oral PrEP due to stigma, discretion, difficulty attending frequent refill visits, or pill burden are underrepresented in PrEP clinical trials, yet they are likely the primary target population for uptake of new PrEP modalities, as current oral PrEP coverage is low [1]. Further, individuals participating in PrEP studies may differ from the general population in that they may be more interested in the PrEP modality evaluated in the study in which they are participating. For example, in a study that assessed preferences among participants in the DVR efficacy trial (MTN-020/ASPIRE), 94% of participants selected the vaginal ring as their most preferred LA PrEP product [41]. However, in the TRIO study, in which women were assigned to use all three of injectable, oral, and ring formulations, only 12% of individuals most preferred the vaginal ring [11]. Future studies should consider investigating PrEP preferences among oral PrEP-naïve individuals not participating in PrEP studies.

Additionally, the majority of included studies were conducted in South Africa, Uganda, Zimbabwe, and Kenya, with little representation from Western and Central Africa. Given the substantial HIV incidence and cultural differences, more research is needed to understand PrEP preferences in these regions.

Finally, results are largely stated preferences regarding hypothetical uptake and not observed use. Cabotegravir is the only LAI currently available, and 18 of 36 studies assessing LAIs were published before cabotegravir was approved for use as PrEP and before lenacapavir studies demonstrated high efficacy [72]. Similarly, implantable PrEP is still in development. In both cases experience with injectable or implantable contraceptives can be a useful proxy for experience with that modality for PrEP products [49]. However, participants analogizing injectables and implants with hormonal contraceptives often feared side effects similar to contraceptives, even though the side effect profile for ARVs is milder than that for contraceptive hormones. Further research will be needed to understand preference for long-acting modalities outside of clinical trials as LA PrEP becomes widely available.

## Conclusion

Long-acting PrEP formulations are highly acceptable across demographic subpopulations in ESA and can increase PrEP coverage to meet global targets for HIV prevention. Overall, injectable PrEP was most preferred followed by biodegradable implants, with product duration playing the most salient role in preferences. The intravaginal ring was the least preferred LA product but still more preferred than daily oral PrEP. There was significant interest in on-demand oral dosing among participants with less frequent HIV exposures. Further research will be needed to understand real-world preference long-acting modalities become widely available.

## Electronic supplementary material

Below is the link to the electronic supplementary material.

Supplementary Material 1: **Table S1**. Search strings. **Table S2**. Quality assessment checklist

## Data Availability

All data generated or analysed during this study are included in this published article and its supplementary information file.

## Abbreviations

PrEP: Pre-exposure prophylaxis
ESA: Eastern and Southern Africa
LAI: Long-acting injectable
DVR: Dapivirine ring
ARV: Antiretroviral
AGYW: Adolescent girls and young women
ABYM: Adolescent boys and young men
FSW: Female sex worker
MSM: Men who have sex with men
CAB-LA: Cabotegravir long-acting
